# Comparative analysis of arthroscopic technique for anterior talofibular and calcaneofibular ligament reconstruction *versus* open modified brostrom-gould procedure in chronic lateral ankle instability management

**DOI:** 10.1186/s13018-024-04800-1

**Published:** 2024-05-28

**Authors:** Gang Hong, XiaoChuan Kong, Le Zhang, YinFeng Zheng, Ning Fan, Lei Zang

**Affiliations:** Department of Orthopedics, Beijing Chaoyang Hospital, Capital Medical University, NO. 5 Jingyuan Road, Shijingshan District, Beijing, 100043 China

**Keywords:** Chronic lateral ankle instability, Anterior talofibular ligament reconstruction, Calcaneofibular ligament reconstruction, Modified brostrom procedure, Orthopedic surgical outcomes

## Abstract

**Background:**

Chronic Lateral Ankle Instability (CLAI) is a common condition treated using either Anterior Talofibular and Calcaneofibular Ligament (ATFL and CFL) reconstruction or Modified Brostrom Procedure (MBP). However, the comparative efficacy of these approaches is not well-studied.

**Methods:**

In this study, clinical data were retrospectively collected from 101 patients diagnosed with CLAI who underwent either ATFL and CFL reconstruction (*n* = 51) or the MBP (*n* = 50). Patients were comparable in terms of age, sex, Body Mass Index (BMI), post-injury duration, preoperative American Orthopedic Foot and Ankle Society (AOFAS) score, Karlsson score, Visual Analog Score (VAS), Anterior Talar Translation, and Talar Tilt Angle.

**Results:**

The post-operative measures showed no significant differences in AOFAS Score, Karlsson Score, and VAS between both treatment groups. However, patients who underwent ATFL and CFL reconstruction showed significantly lower follow-up Anterior Talar Translation (mean = 4.1667 ± 1.3991 mm) and Talar Tilt Angle (mean = 5.0549 ± 1.6173°) compared to those who underwent MBP. Further, patients treated with ATFL and CFL reconstruction experienced a significantly longer postoperative recovery time (median = 6 weeks) compared to MBP (median = 3 weeks).

**Conclusions:**

Although both therapeutic techniques were generally effective in treating CLAI, the ATFL and CFL reconstruction approach delivered superior control of Anterior Talar Translation and Talar Tilt Angle. However, its longer recovery time merits further study to optimize the balance between therapeutic efficacy and recovery speed.

## Introduction

Chronic Lateral Ankle Instability (CLAI) presents a significant clinical challenge in orthopedic and sports medicine, affecting a considerable portion of the population engaged in physical activities [1]. This condition, characterized by recurrent ankle sprains and a persistent sensation of instability, often leads to a diminished quality of life and a reduced ability to participate in athletic or daily activities [2]. The underlying pathology typically involves the impairment or insufficiency of the lateral ankle ligaments, particularly the Anterior Talofibular Ligament (ATFL) and the Calcaneofibular Ligament (CFL), which have the function of limiting plantarflexion and ankle inversion and contributing greatly to the overall stability of the ankle joint [3]. As a result, the ankle joint experiences excessive inversion and anterior translation, predisposing it to recurrent injuries and progressive instability [4]. Given its prevalence and impact, effective management of CLAI is crucial for restoring ankle stability, function, and preventing further joint degeneration.

The management of CLAI traditionally revolves around conservative and surgical interventions [5]. Conservative management like massage, traction, hot and cold water therapy, is the preferred approach for acute lateral ankle ligament injuries, with surgery reserved for cases of treatment failure [6]. Operative options include anatomic repair, anatomic reconstruction with autograft or allograft, and tenodesis procedures [7]. Ankle arthroscopy can be used as an approach for anatomic repair (Corte Real 2009) or reconstruction and to identify and treat associated intra-articular conditions [8]. Tenodesis techniques are not recommended due to suboptimal long-term results [9]. The Open Modified Brostrom Procedure (MBP) and the reconstruction of ATFL and CFL are two prevalent surgical interventions for CLAI. If the ATFL remnant is not viable, MBP procedure without ATFL suturing can still be performed instead of a more complex lateral ligament reconstruction [10]. . The MBP, a procedure that tightens and reinforces the existing ligaments, has been a gold standard in the treatment of this condition for several years [11]. In contrast, ATFL and CFL reconstruction involves the replacement of these ligaments with grafts to restore ankle stability [12]. The choice between these procedures is often influenced by patient-specific factors, the severity of ligamentous damage, and surgeon preference [13]. Despite the widespread application of these procedures, there exists a notable gap in the comparative evaluation of their efficacy and outcomes, particularly in the context of long-term joint stability and recovery times. Prior research has primarily focused on the outcomes of these procedures in isolation, evaluating aspects such as postoperative stability, pain, function, and patient satisfaction. However, these studies often lack direct comparisons between the two methods, leaving a critical question unanswered: Which surgical approach offers superior outcomes for patients with CLAI?

Recognizing this gap, the current study aims to provide a comparative analysis of these two prevalent surgical techniques during anatomical arthroscopic reconstruction. By retrospectively examining clinical data from 101 patients diagnosed with CLAI and treated with either ATFL and CFL reconstruction or MBP, this research provides a unique opportunity to compare these approaches. The study’s comprehensive evaluation includes various metrics such as the American Orthopedic Foot and Ankle Society (AOFAS) score, Karlsson score, Visual Analog Score (VAS), Anterior Talar Translation, and Talar Tilt Angle. This holistic approach ensures a multifaceted assessment of the outcomes, encompassing both functional and anatomical aspects.

## Methods

### Study design and population

This study was a retrospective comparative analysis conducted at a tertiary orthopedic center. We included patients with CLAI who underwent either ATFL and CFL Reconstruction or MBP between January 2019 and December 2023. CLAI was diagnosed based on clinical evaluation and magnetic resonance imaging (MRI) findings. Inclusion criteria were adults aged 18–45 years with a history of recurrent ankle sprains and failed conservative treatment for at least six months. Patients with a history of previous ankle surgery, concomitant ankle fractures, systemic inflammatory diseases, or neuromuscular disorders were excluded from the study.

### Surgical procedures

Two surgical procedures were analyzed. ATFL and CFL Reconstruction procedure involved the reconstruction of both the ATFL and CFL using gracilis autografts. The grafts were anchored to the fibula and talus with interference screws. The peroneal tendon sheath was incised, and the scope was introduced. The scope was placed anteriorly to the peroneal brevis tendon and carefully pushed up dis-tally. The peroneal tendons were inspected for lesions. The septum of the two separated tendon sheaths was visualized at the distal part of the common tunnel. The CFL was visualized more proximally. The anterior part was sutured to the Jugger Knot, which was used to pull the graft into the talar tunnel. Afterwards, this was fixed with an All-thread interference screw 5.5 × 15 mm (Biomet, Warsaw, Indiana). The Toggle Loc allowed the new ATFL to be tightened by an additional 5 mm, if necessary. The other end was pulled through the calcaneus and fixed with a Gentle Thread interference screw of 7 × 25 mm (Biomet, Warsaw, Indiana). During the fixation, the ankle joint was held in a valgus position. Ankle surgery with the open modified Broström procedure (MBP) involved the repair of the ATFL and CFL with sutures and reinforcement with local tissue. The retinaculum was imbricated to enhance lateral stability. All surgeries were performed by the same surgical team using a standardized technique. Postoperative rehabilitation protocols were similar for both groups.

### Data collection

Clinical data were collected retrospectively from medical records. Baseline data were collected, including age, sex, body mass index (BMI), surgery duration of each kind of procedure, duration of symptoms post-injury, preoperative AOFAS score (assessing pain, function, and alignment), Karlsson score (evaluating ankle joint stability), VAS score (measuring pain intensity), anterior talar translation (evaluated through stress radiographs), and talar tilt angle (evaluated through stress radiographs). Follow-up data were collected at 12 months postoperatively, which included AOFAS score, Karlsson score, VAS score, anterior talar translation (the talus slides forward, squeezing the front of the ankle and limiting the range of the hook), talar tilt angle (the angle between the parietal talus and the tibial dome), and postoperative recovery time of complete relief of pain and swelling. We rated the severity of ligamentous injury as the typical grading scale of a ligament injury consists of *grade I*, which describes minor elongation with microdamage; *grade II*, more involved stretching and insult but without compromised structural integrity; and *grade III*, complete rupture [14].

### Statistical analysis

Data were analyzed using SPSS version 27. Continuous variables were presented as mean ± standard deviation (SD) or median with interquartile range (IQR), and categorical variables as numbers and percentages. The normality was assessed using the Shapiro-Wilk test, while homogeneity of variances was assessed using Levene’s test. The Mann-Whitney U test was employed for non-normally distributed data, the Student’s t-test for normally distributed data with homogeneity of variances, and Welch’s t-test for normally distributed data without homogeneity of variances. Categorical data were analyzed using the Chi-square test or Yates’ correction as appropriate. A *p*-value of less than 0.05 was considered statistically significant.

### Ethical considerations

The study was conducted following the Declaration of Helsinki and was approved by the Institutional Review Board. Patient confidentiality was maintained throughout the study, and all data were anonymized before analysis.

## Results

### Baseline characteristics of study participants

The study involved 101 patients with CLAI, with 51 undergoing ATFL and CFL Reconstruction and 50 receiving the MBP (Table 1). Baseline demographic and clinical characteristics were comparable between the two groups. The median age was 28 years in the ATFL and CFL group and 28.5 years in the MBP group (*P* = 0.385). Gender distribution was also similar, with 56.9% females in the ATFL and CFL group and 62% in the MBP group (*P* = 0.599). The median BMI was 25.01 kg/m² for the ATFL and CFL group and 24.03 kg/m² for the MBP group, showing no significant difference (*P* = 0.129). Additionally, the duration of post-injury was similar across the groups, with a median of 19 months for ATFL and CFL and 18.5 months for MBP (*P* = 0.678). Preoperative clinical scores including AOFAS, Karlsson, and VAS scores, as well as anterior talar translation and talar tilt angle, were comparable between the groups, indicating a balanced baseline for both surgical techniques.

**Table 1:** Baseline data of chronic lateral ankle instability patients

Characteristics	ATFL and CFL Reconstruction	Modified Brostrom Procedure	*P* value
n	51	50	
Age, median (IQR)	28 (24.5, 31)	28.5 (26, 32)	0.385
Sex, n (%)			0.599
Female	29 (56.9%)	31 (62%)	
Male	22 (43.1%)	19 (38%)	
BMI (kg/m2), median (IQR)	25.01 (23.01, 26.075)	24.03 (20.332, 26.018)	0.129
Post-injury duration (months), median (IQR)	19 (15, 23.5)	18.5 (15.25, 23)	0.678
Preoperative AOFAS score, mean ± sd	56.314 ± 5.4936	56.98 ± 8.726	0.648
Preoperative Karlsson score, mean ± sd	61.059 ± 10.029	61.56 ± 9.6767	0.799
Preoperative VAS score, median (IQR)	4 (3, 4)	4 (3, 5)	0.288
Preoperative anterior talar translation (mm), mean ± sd	10.404 ± 2.1716	10.628 ± 4.834	0.765
Preoperative talar tilt angle (°), mean ± sd	10.722 ± 3.6253	12.03 ± 3.2469	0.059

### Outcome measures at follow-up

At the follow-up, both groups showed significant improvement in clinical outcomes, but with different degrees of efficacy in certain parameters. The follow-up AOFAS score was 80.745 ± 3.7939 in the ATFL and CFL group and 80.92 ± 8.0884 in the MBP group, indicating no significant difference in overall foot and ankle function (*P* = 0.890). The Karlsson score, which assesses ankle joint performance, was 87.961 ± 9.7344 for ATFL and CFL and 90.54 ± 5.6142 for MBP, showing a trend towards better performance in the MBP group, although not statistically significant (*P* = 0.106). In terms of pain assessment through the VAS score, both groups demonstrated improvement with no significant difference in the distribution of scores (*P* = 0.224) (Table 2).

**Table 2:** Follow-up data of chronic lateral ankle instability patients

Characteristics	ATFL and CFL Reconstruction	Modified Brostrom Procedure	*P* value
n	51	50	
Follow-up AOFAS score, mean ± sd	80.745 ± 3.7939	80.92 ± 8.0884	0.890
Follow-up Karlsson score, mean ± sd	87.961 ± 9.7344	90.54 ± 5.6142	0.106
Follow-up VAS score, n (%)			0.224
0	19 (37.3%)	15 (30%)	
1	12 (23.5%)	18 (36%)	
2	20 (39.2%)	15 (30%)	
3	0 (0%)	2 (4%)	
Follow-up anterior talar translation (mm), mean ± sd	4.1667 ± 1.3991	6.69 ± 1.5143	< 0.001
Follow-up talar tilt angle (°), mean ± sd	5.0549 ± 1.6173	6.606 ± 2.0376	< 0.001
Postoperative recovery time (weeks), median (IQR)	6 (4, 7)	3 (2.25, 4)	< 0.001

### Comparative analysis of anatomical outcomes

The anatomical outcomes revealed significant differences between the two surgical techniques. The mean postoperative anterior talar translation was significantly lower in the ATFL and CFL group (4.1667 ± 1.3991 mm) compared to the MBP group (6.69 ± 1.5143 mm), with a *P*-value of < 0.001. Similarly, the follow-up talar tilt angle was significantly reduced in the ATFL and CFL group (5.0549 ± 1.6173°) compared to the MBP group (6.606 ± 2.0376°), also with a *P*-value of < 0.001. These findings indicate a more effective restoration of anatomical alignment in the ATFL and CFL reconstruction group.

### Postoperative recovery time

The median postoperative recovery time was significantly different between the two groups. Patients undergoing ATFL and CFL Reconstruction reported a longer recovery time with a median of 6 weeks (IQR: 4–7 weeks) compared to 3 weeks (IQR: 2.25-4 weeks) in the MBP group (*P* < 0.001). This suggests a faster return to normal activities following MBP.

## Discussion

CLAI is a multifactorial condition that can result from a single severe ankle sprain or multiple minor injuries. These events lead to the stretching or tearing of the ATFL and CFL, causing mechanical instability and functional impairment [15]. The MBP, first described by Brostrom in 1966, is a well-established technique involving the imbrication and repair of the injured ligaments [16]. On the other hand, ligament reconstruction with grafts is a relatively newer approach that has gained traction in cases where the native ligaments are deemed insufficient for repair [17].

Our research showed no significant differences in the AOFAS Score, Karlsson Score, and VAS between the ATFL and CFL reconstruction group and the MBP group. This result is consistent with prior studies that have also reported comparable outcomes in functional scores following different surgical techniques for CLAI. For instance, a study by Tong Su et al. observed similar improvements in patient-reported outcome measures between anatomic reconstruction using the autologous gracilis tendon and MBP [18, 19].

Notably, our study revealed that ATFL and CFL reconstruction provided significantly better control of Anterior Talar Translation and Talar Tilt Angle compared to MBP. This finding is particularly interesting as it aligns with the biomechanical goals of CLAI surgery, which aim to restore ankle stability. A study by Yeqiang Luo and colleagues also noted improved biomechanical outcomes in patients undergoing ligament reconstruction, corroborating our findings [20]. These improved biomechanical parameters might be attributed to the more anatomical repair or reconstruction of the ligaments in ATFL and CFL reconstruction compared to the non-anatomical approach of MBP. A study by Gang Zeng and colleagues suggested that open Broström-Gould repair and all-arthroscopic anatomical repair of the ATFL have comparable therapeutic efficacy for chronic lateral ankle instability. The arthroscopic surgery had a smaller incision, while the open Broström-Gould had a shorter surgery duration and lower cost [21].

However, the longer recovery time associated with ATFL and CFL reconstruction is a significant consideration. Our study found a median recovery time of 6 weeks for ATFL and CFL reconstruction, compared to 3 weeks for MBP. This aspect of recovery is critical in clinical decision-making, especially for athletes or individuals whose occupations demand quick return to activity. Previous research by Zong-Chen Hou et al. highlighted the importance of recovery time in surgical decision-making, noting that longer recovery periods could negatively impact patients’ quality of life and economic situations [22]. Therefore, while the biomechanical advantages of ATFL and CFL reconstruction are clear, the extended recovery period poses a challenge that warrants further investigation. The balance between therapeutic efficacy and recovery speed is a critical aspect of CLAI treatment. Our study suggests that while ATFL and CFL reconstruction may offer superior biomechanical stability, the longer recovery period is a significant trade-off. This is a complex decision matrix for both patients and surgeons, as the choice of procedure may depend on individual patient needs, lifestyle, and professional demands. Future research could focus on modifying surgical techniques or postoperative rehabilitation protocols to shorten the recovery time associated with ATFL and CFL reconstruction without compromising its biomechanical benefits. In addition, one of the advantages of open MBG surgery compared to other surgeries is its lower learning curve and faster surgical speed. Proficient surgical procedures and faster surgical speed may also have a positive impact on the prognosis of patients.

This study is not without its limitations that should be acknowledged. First, our study design was retrospective in nature, which inherently involves potential selection bias and limits our ability to establish causal relationships. Further, due to the retrospective design, we relied on medical records and may not have access to all relevant information or factors influencing patient outcomes. Second, after determining the type I and type II error values, minimal clinically relevant difference and the variance of their own study, our calculated sample size, while fairly large, does not allow for subgroup analysis. It thus could not provide further insight into the effectiveness of the procedures in specific patient populations. Third, our follow-up period was not long-term, and therefore, we could not assess the durability of the surgical procedures over an extended period. Last, as we have not included the power analysis to determine the sample size in this study, we cannot make specific conclusions with no statistically significant difference. While these are crucial in understanding the patients’ perception of their functionality, they are inherently subjective and potentially influenced by numerous variables outside of the surgical procedure. Lastly, we did not control for potential confounding factors such as the varying expertise of the surgeons and the individual patient’s rehabilitation compliance, both of which could significantly affect the observed outcomes. Therefore, the findings of this study should be interpreted with these limitations in mind and validated with additional randomized controlled trials with more extended follow-up periods.

In conclusion, both the ATFL and CFL reconstruction and the MBP were generally effective in treating CLAI, as evidenced by comparable AOFAS Score, Karlsson Score, and VAS outcomes. However, the ATFL and CFL reconstruction approach demonstrated superior control of specific ankle stability measures, albeit with a longer recovery time. This highlights the importance of considering both clinical efficacy and recovery duration when choosing the optimal treatment approach for CLAI. Further research is warranted to refine treatment strategies and enhance patient outcomes in this context.

## Data Availability

No datasets were generated or analysed during the current study.
